# Energy efficiency of cookie residue and its effects on broiler performance

**DOI:** 10.3389/fvets.2025.1587576

**Published:** 2025-05-15

**Authors:** Lidiane Rosa Custódio, Maria do Carmo Mohaupt Marques Ludke, Ana Carolina Ferreira dos Santos, Thaysa Rodrigues Torres, Jussiede Silva Santos, Julia da Silva Barros, Jorge Vitor Ludke, Dayane Albuquerque da Silva, Apolônio Gomes Ribeiro, Júlio Cézar dos Santos Nascimento, Carlos Bôa-Viagem Rabello, Arlei Coldebella, Lucas Rannier Ribeiro Antonino Carvalho

**Affiliations:** ^1^Universidade Federal Rural de Pernambuco, Animal Science Department, Recife, PE, Brazil; ^2^Universidade Federal Rural de Pernambuco, Academic Unit of Serra Talhada, Serra Talhada, PE, Brazil; ^3^Embrapa Swine and Poultry, Concórdia, SC, Brazil; ^4^Universidade Federal da Paraíba, Animal Science Department, Areia, PB, Brazil; ^5^Karolinska Institutet, Department of Physiology and Pharmacology, Stockholm, Sweden

**Keywords:** alternative feed, blood parameters, carcass, feed-to-gain ratio, food residue

## Abstract

This study evaluated the energy value of cookie residue in broiler diets through a metabolism trial and determined the optimal inclusion level based on performance, carcass characteristics, and blood parameters. Two experiments were conducted using a completely randomized design. In the metabolism trial, 60 broilers (14 days old) were assigned to two treatments: a control diet and a diet with 30% cookie residue replacing the reference feed, with six replicates of five birds each. In the performance trial, 450 1-day-old broilers were assigned to five treatments (0%, 10%, 20%, 30%, and 40% cookie residue), with six replicates of 15 birds per treatment, evaluated at 7, 21, 35, and 42 days of age. The cookie residue showed an apparent metabolizable energy (AME) of 3,959 Kcal kg^−1^ and nitrogen-corrected AME (AMEn) of 3,480 Kcal kg^−1^. Performance results indicated that during the 1–7, 8–21, and 1–21 day periods, birds fed diets with cookie residue performed better than those on the control diet. However, no significant differences in overall performance or carcass characteristics were observed from 1 to 42 days, except for an increase in abdominal fat, a decrease in empty gizzard weight, and elevated blood cholesterol and creatinine levels—though all values remained within physiological norms. These findings suggest that cookie residue can be included in broiler diets at levels up to 40% without compromising performance or carcass quality.

## 1 Introduction

Sweet and salt cookie production in Brazil is the fourth highest in the world, with 1,157,051 tons of cookies sold, generating 14.332 billion reais in income. According to decree no 12/78 of the National Commission of Norms and Standards for Food (1), a cookie is defined as a product obtained through the kneading and cooking of dough prepared with flour, starch, and other food substances, fermented or not. Among the most produced salt cookies, 253,460 tons were sold, with a market value of 2.275 billion Brazilian reais (2). These cookies are made from wheat flour, hydrogenated vegetable fat, starch, malt extract, salt, whey, inverted sugar, sugar, biological yeast, sodium bicarbonate, soy lecithin, and water. Sweet and salt cookies are produced through the fermentation of the mixture of ingredients. This production is divided into two phases: the sponge phase, in which water and yeast are mixed with part of the flour and left to ferment to develop flavor and leavening; and the dough phase, where the remaining ingredients such as bicarbonate and fat are added to complete the mixture, resulting in a consistent dough ready for shaping and baking.

During cookie manufacturing, material losses occur at several processing stages. Garcia et al. (3) analyzed total production losses and found that for each daily production of 70 tons of sweet and salt cookies, around 12.96 tons (18.51% of the production) are lost in the process, primarily due to baking rejects (burnt or raw cookies outside the specification standard or that break; 5.51% of total losses), packaging (breaks and cracks in packaging; 10% of total losses), and losses due to sweeping in packaging (cookies that fall to the ground during this process and are sold as animal feed; 3% of total losses).

In recent years, there has been an increase in the amount of food residue due to the growth of the urban population and, consequently, the high demand for cookies. This has raised concerns among inspection bodies, as industrial residue that is disposed of improperly can cause environmental pollution. The material lost during production must be discarded, as it is unsuitable for human consumption, but it is rich in starch and fat and can therefore be added to animal feed. According to Rostagno et al. (4), cookie residue is 92.5% dry matter (DM), 8.69% crude protein (CP), 8.28% ether extract (EE), 1.71% crude fiber (CF), and 1.31% mineral matter (MM), and has metabolizable energy of 4,010 Kcal kg^−1^. This composition may vary according to the ingredients and/or the processing to which it is subjected. Costa (5) performed experiments in which broilers were fed cookie residue and obtained apparent metabolizable energy (AME) and metabolizable energy corrected for nitrogen (AMEn) of 3,959 ± 0.458 and 3,480 ± 0.399 Kcal kg^−1^, respectively. For poultry, these values are higher than those for corn.

The inclusion of cookie residue in this study is justified by its origin as a by-product of the food industry, which, due to not meeting commercial standards for human consumption, is commonly discarded. This residue stands out for its high energy content, mainly due to its composition rich in carbohydrates and lipids, and is also widely available. Its use in animal feed adds value to a material that would otherwise be wasted, contributing to the reduction of industrial waste and promoting sustainability and circular economy principles within the agri-food sector.

This study aimed to evaluate the energy and nutritional value of cookie residue (Maizena cookies) and the optimal level of inclusion in broiler diets, considering the performance of the animals, carcass characteristics, and blood profile.

## 2 Materials and methods

The present study comes from the project approved by the Ethics Committee on the Use of Animals of the Federal University of Pernambuco (UFPE) under number 087/2016.

### 2.1 Animals and experimental design

Two experiments were carried out with male Cobb 500^®^ broilers in the experimental facilities of UFRPE, located in the municipality of Recife, PE (8°04′03′'S, 34°55′00^′′^W). The first experiment was a metabolism assay to assess the AME and AMEn of the cookie residue. The second experiment was conducted to evaluate the performance, carcass characteristics, and blood parameters of broilers fed a diet with cookie residue.

The metabolism experiment used 60 broilers aged 14–22 days and with an average weight of 481.5 ± 0.50 g. The birds were distributed in a completely randomized design with two treatments: a reference diet (Table 1) based on corn and soybean meal, and a test diet with 30% cookie residue replacing part of the reference diet. The cookie residue replaced a portion of the corn and soybean meal in the reference diet, altering the carbohydrate and lipid composition of the diet. There were six replicates, with five birds per experimental unit.

**Table 1:** Nutritional composition and calculated energy value of the reference diet used in the metabolism test.

**Ingredients**	**Amount (%)**	**Nutritional composition and energy value (%)**	
Corn	55.56	AMEn^*^ (Kcal kg^−1^)	3,050
Soybean meal 45%	36.97	Crude protein	21.2
Soy oil	3.55	Calcium	0.84
Dicalcium phosphate	1.55	Available phosphorus	0.40
Limestone	0.92	Sodium	0.21
Common salt	0.48	Ether extract	6.16
L-Lysine HCl 78.8%	0.23	Crude fiber	3.0
DL-Methionine 99%	0.30	Digestible amino acids	
L-Threonine 98.5%	0.08	Lysine	1.217
Vitamin supplement^a^	0.15	Methionine	0.580
Mineral supplement^b^	0.12	Methionine + cystine	0.876
Choline chloride 60%	0.10	Threonine	0.791
Total	100	Tryptophan	0.284

^*^Apparent metabolizable energy corrected for nitrogen balance (AMEn). ^a^Vitamin premix has the following levels per kg of product: vitamin A (10,000,000 IU), vitamin D3 (2,000,000 IU), vitamin E (20,000 mg), vitamin K3 (4,000 mg), vitamin B1 (1,880 mg), vitamin B2 (5,000 mg), vitamin B6 (2,000 mg), vitamin B12 (10,000 μg), niacin (30,000 mg), pantothenic acid (13,500 mg), and folic acid (500 mg). ^b^Mineral premix has the following levels per kilogram of product: selenium (360 mg), zinc (110,000 mg), iodine (1,400 mg), copper (20,000 mg), manganese (156,000 mg), iron (96,000 mg), and antioxidant (100,000 mg).

The birds were housed in cages measuring 1.00 × 0.50 × 0.50 m, equipped with trough-type feeders and cup-type drinkers, with support for collection trays covered with plastic sheets. The experimental period began at 14 days of age, with 4 days for adaptation to the experimental diets and 4 days for total excreta collection. One percent ferric oxide was added to the experimental rations to mark the beginning and end of the collection period.

Two collections were performed daily (at 8 am and 2 pm). The collected material was weighed and preserved in labeled plastic bags and stored at −20°C. At the end of the experimental period, the samples were thawed, homogenized, weighed, and pre-dried in a forced ventilation oven at 55°C for 72 h. The dried excreta were ground in a ball mill, and the experimental rations were ground in a hammer mill with a 1.00-mm mesh sieve. The ground materials were sent to the laboratory to determine the dry matter (DM), crude protein (CP), and gross energy (GE) contents according to the methodology described by Detmann et al. (6). Then, the apparent metabolization coefficients of dry matter (AMCDM), crude protein (AMCCP), and gross energy (AMCGE) were calculated. The AME and AMEn were obtained by applying the equations described by Matterson et al. (7).

In the performance experiment, 450 broilers were used from 1 to 42 days. They were distributed in a completely randomized design consisting of five treatments (control diet based on corn and soybean meal and four diets formulated with inclusion of 10, 20, 30, and 40% cookie residue) and six replicates, with 15 birds per experimental unit. The rations were isoproteic and isoenergetic and formulated to meet the requirements recommended by Rostagno et al. (4) (Tables 2, 3). Water and feed (mash) were provided *ad libitum*. The dietary program consisted of four broiler phases, namely, the pre-starter phase (1–7 days), starter phase (8–21 days), growth phase (22–35 days), and finisher phase (36–42 days).

**Table 2:** Nutritional composition of the diets used for the pre-starter (days 1–7) and starter (days 8–21) phases.

**Ingredient (%)**	**Cookie residue inclusion levels (%)**									
	**1 to 7 days**					**8 to 21 days**				
	**0**	**10**	**20**	**30**	**40**	**0**	**10**	**20**	**30**	**40**
Corn	54.57	45.21	35.85	26.49	17.14	57.01	47.66	38.30	28.94	19.59
Soybean meal 45%	38.75	38.41	38.06	37.72	37.38	35.90	35.56	35.21	34.87	34.53
Cookie residue	0.0	10.0	20.0	30.0	40.0	0.0	10.0	20.0	30.0	40.0
Soy oil	2.37	2.08	1.79	1.50	1.22	3.24	2.96	2.67	2.38	2.09
Dicalcium phosphate	1.90	1.90	1.90	1.90	1.90	1.55	1.56	1.56	1.56	1.56
Limestone	0.91	0.91	0.90	0.89	0.88	0.94	0.94	0.93	0.92	0.91
Common salt	0.51	0.46	0.42	0.38	0.33	0.48	0.44	0.39	0.35	0.31
L-Lysine HCl 78.8%	0.29	0.30	0.32	0.33	0.35	0.24	0.25	0.27	0.28	0.30
DL-methionine 99%	0.36	0.37	0.37	0.38	0.39	0.31	0.32	0.32	0.33	0.33
L-Threonine 98.5%	0.12	0.13	0.15	0.16	0.18	0.08	0.09	0.11	0.12	0.14
Premix^a^	0.12	0.12	0.12	0.12	0.12	0.12	0.12	0.12	0.12	0.12
Coccidiostatic	0.10	0.10	0.10	0.10	0.10	0.10	0.10	0.10	0.10	0.10
**Calculated composition (%)**										
AME (Kcal kg^−1^)	2,960	2,960	2,960	2,960	2,960	3,050	3,050	3,050	3,050	3,050
Crude protein	22.40	22.40	22.40	22.40	22.40	21.20	21.20	21.20	21.20	21.20
Calcium	0.92	0.92	0.92	0.92	0.92	0.84	0.84	0.84	0.84	0.84
Available phosphorus	0.47	0.47	0.47	0.47	0.47	0.40	0.40	0.40	0.40	0.40
Sodium	0.22	0.22	0.22	0.22	0.22	0.21	0.21	0.21	0.21	0.21
Ether extract	5.25	5.49	5.73	5.97	6.20	6.19	6.42	6.66	6.90	7.14
Crude fiber	3.00	2.98	2.97	2.96	2.95	2.89	2.88	2.86	2.86	2.84
**Digestible amino acids (%)**										
Lysine	1.324	1.324	1.324	1.324	1.324	1.217	1.217	1.217	1.217	1.217
Methionine + cystine	0.953	0.953	0.953	0.953	0.953	0.876	0.876	0.876	0.876	0.876
Threonine	0.861	0.861	0.861	0.861	0.861	0.791	0.791	0.791	0.791	0.791
Tryptophan	0.252	0.253	0.254	0.256	0.252	0.237	0.238	0.239	0.240	0.242
**Analyzed composition (%)**										
Dry matter	88.91	89.00	89.33	89.85	90.74	87.70	89.19	89.85	89.42	89.77
Crude protein	22.79	22.60	22.42	22.41	22.19	21.63	21.51	21.39	21.34	21.17
Ether extract	5.12	5.42	5.72	5.98	6.17	6.20	6.33	6.68	6.89	7.12
Crude fiber	3.09	3.01	2.96	2.89	2.90	2.91	2.87	2.85	2.86	2.83
Ashes	4.58	4.49	4.11	4.26	4.16	4.39	4.24	4.15	4.34	4.26
MGD (μm)	768.3	753.4	735.8	621.0	610.4	804.1	782.0	769.9	743.0	699.2
Density (kg L^−1^)	0.463	0.442	0.438	0.425	0.409	0.483	0.449	0.433	0.429	0.408

Apparent metabolizable energy corrected for nitrogen (AMEn); mean geometric diameter (MGD). ^a^Guaranteed levels per kilogram of vitamin–mineral supplement: vitamin A (5,000,000 IU); folic acid (150 mg), pantothenic acid (8,000 mg), biotin (40 mg), niacin (18 g), vitamin B12 (6,500 μg), vitamin B2 (2,000 mg), vitamin B6 (250 mg), vitamin D3 (1,600,000 IU), vitamin K3 (1,000 mg), copper (1,400 mg), iron (6,000 mg), iodine (915 mg), manganese (17 g), selenium (800 mg), and zinc (33 g).

**Table 3:** Nutritional composition of the diets used in the growth (days 22–35) and finisher (days 36–42) phases.

**Ingredient (%)**	**Cookie residue inclusion levels (%)**									
	**22 to 35 days**					**36 to 42 days**				
	**0**	**10**	**20**	**30**	**40**	**0**	**10**	**20**	**30**	**40**
Corn	60.00	50.65	41.29	31.93	22.58	64.26	54.90	45.54	36.19	26.83
Soybean meal 45%	32.32	31.98	31.63	31.29	30.95	28.45	28.10	27.76	27.42	27.07
Cookie residue	0.0	10.0	20.0	30.0	40.0	0.0	10.0	20.0	30.0	40.0
Soy oil	4.17	3.88	3.58	3.30	3.01	4.09	3.80	3.51	3.22	2.94
Dicalcium phosphate	1.33	1.34	1.33	1.34	1.34	1.12	1.12	1.13	1.17	1.13
Limestone	0.89	0.88	0.87	0.87	0.86	0.80	0.79	0.78	0.77	0.77
Common salt	4.6	4.1	3.7	3.3	2.8	4.4	4.0	3.6	3.1	2.7
L-Lysine HCl 78.8%	2.4	2.5	2.7	2.8	3.0	2.7	2.8	3.0	3.1	3.3
DL-Methionine 99%	2.9	3.0	3.0	3.1	3.1	2.7	2.7	2.7	2.9	3.0
L-Threonine 98.5%	0.7	0.9	1.0	1.2	1.4	0.8	0.9	1.0	1.2	1.4
Premix^a^	1.2	1.2	1.2	1.2	1.2	1.2	1.2	1.2	1.2	1.2
Coccidiostatic	1.0	1.0	1.0	1.0	1.0	1.0	1.0	1.0	1.0	1.0
**Calculated composition (%)**										
AME (Kcal/kg)	3,150	3,150	3,150	3,150	3,150	3,200	3,200	3,200	3,200	3,200
Crude protein	19.80	19.80	19.80	19.80	19.80	18.40	18.40	18.40	18.40	18.40
Calcium	0.76	0.76	0.76	0.76	0.76	0.66	0.66	0.66	0.66	0.66
Available phosphorus	0.35	0.35	0.35	0.35	0.35	0.31	0.31	0.31	0.31	0.31
Sodium	0.20	0.20	0.20	0.20	0.20	0.20	0.20	0.20	0.20	0.20
Ether extract	7.17	7.41	7.65	7.88	8.12	7.21	7.45	7.68	7.92	8.16
Crude fiber	2.75	2.74	2.73	2.72	2.71	2.63	2.62	2.61	2.60	2.59
**Digestible amino acids (%)**										
Lysine	1.131	1.131	1.131	1.131	1.131	1.060	1.060	1.060	1.060	1.060
Methionine + cystine	0.826	0.826	0.826	0.826	0.826	0.774	0.774	0.774	0.774	0.774
Threonine	0.735	0.735	0.735	0.735	0.735	0.689	0.689	0.689	0.689	0.689
Tryptophan	0.217	0.218	0.220	0.221	0.222	0.197	0.198	0.200	0.201	0.203
**Analyzed composition (%)**										
Dry matter	88.25	88.54	89.52	89.61	89.70	89.09	87.42	89.04	89.57	89.09
Crude protein	19.90	19.71	19.75	19.82	19.74	18.47	18.41	18.50	18.41	18.49
Ether extract	7.19	7.41	7.63	7.89	8.11	7.23	7.45	7.65	7.92	8.13
Crude fiber	2.74	2.72	2.70	2.68	2.66	2.67	2.59	2.60	2.58	2.55
Ashes	4.33	4.51	4.29	4.27	4.19	4.36	4.29	4.19	4.12	4.10
MGD (μm)	812.7	807.5	789.1	751.2	737.6	815.9	810.0	792.9	755.6	739.8
Density (kg L^−1^)	0.523	0.5126	0.493	0.479	0.476	0.679	0.657	0.632	0.621	0.589

Apparent metabolizable energy corrected for nitrogen (AMEn); mean geometric diameter (MGD). ^a^Guaranteed levels per kilogram of vitamin–mineral supplement: vitamin A (5,000,000 IU); folic acid (150 mg), pantothenic acid (8,000 mg), biotin (40 mg), niacin (18 g), vitamin B12 (6,500 μg), vitamin B2 (2,000 mg), vitamin B6 (250 mg), vitamin D3 (1,600,000 IU), vitamin K3 (1,000 mg), copper (1,400 mg), iron (6,000 mg), iodine (915 mg), manganese (17 g), selenium (800 mg), and zinc (33 g).

Samples of cookie residue as well as the experimental diets were analyzed for the dry matter (DM), crude protein (CP), ether extract (EE), crude fiber (CF), and mineral matter (MM) contents using the methodology described by Detmann et al. (6). The mean geometric diameter (MGD) was analyzed according to the methodology described by Zanotto et al. (8). Density was analyzed with the aid of a glass funnel coupled to a 600-mL beaker and a precision scale; the weight of the sample was determined by dividing the weight by the volume of the beaker.

The performance trial was conducted in an experimental poultry facility with boxes measuring 1.15 × 1.90 × 0.60 m and equipped with tubular feeders and nipple drinkers. There was constant light throughout the experimental period. At the beginning and end of each phase, the birds were weighed to calculate feed consumption, body weight gain, and feed conversion ratio. At the end of the performance experiment, four birds were selected per experimental unit based on average weight: two for blood collection and two for slaughter.

### 2.2 Blood serum biochemistry

Blood was collected by puncturing the ulnar vein in the left wing; approximately 4 mL was collected and transferred to a tube containing ethylenediaminetetraacetic acid (EDTA). The collected material was then centrifuged at 3,800–4,000 rpm for 5 min. The serum (approximately 1 mL) was transferred to an Eppendorf tube, transported to the lab in a Styrofoam container with ice sheets, and stored in a freezer until analysis. The serum samples were thawed and analyzed with a semi-automatic biochemical analyzer (Sinnowa Model SX-3000M^®^) BIOCLIN serological kits, following the manufacturer's protocols. The following parameters were analyzed: liver function measures, including glucose (in mg/dL, Kit Bioclin, Referência k082-2), total glucose (in mg/dL, Kit Bioclin, Referência K082-3), cholesterol (in mg/dL, Kit Bioclin, Referência k083-2), triglycerides (in mg/dL, Kit Bioclin, Referência k117-2), and kidney function measures including urea (in mg/dL, Kit Bioclin, Referência K047-1) and creatinine (in mg/dL, Kit Bioclin, Referência k222-1).

### 2.3 Evaluation of carcass yield, prime cuts, and offal

The birds intended for evaluating carcass yield, prime cuts, and offal were fasted from solids for 6 h. After fasting, they were weighed and then slaughtered. The slaughter stages consisted of stunning, bleeding, scalding, plucking, and evisceration. The carcasses (excluding the head, feet, and viscera) were properly weighed to determine the hot carcass yield. They were then placed in labeled plastic bags and hung in a cold room at 5°C for 24 h. After 24 h, they were removed and weighed to determine the cold carcass weight, and then butchered to determine the yields of the cuts. The hot and cold carcass and organ weights (heart, gizzard, liver, and intestine) are expressed relative to the broiler's fasting weight. The yields of the cuts (breast, thighs, drumsticks, wings, and back) are expressed relative to the cold carcass weight.

### 2.4 Statistical analyses

The evaluated parameters were subjected to statistical analysis with SAS version 9.2 using the PROC GLM procedure for analysis of variance, adopting the F test and an ɑ value of 0.05. The statistical model was


$$\begin{array}{l} yik=\mu +Ti+\;\varepsilon ik \end{array}$$

where y is the performance and carcass evaluation variable, μ is the general average, Ti is the effect of the i^th^ level of cookie residue, and εik is normally distributed random error with zero mean and variance σ2 [εik ~ N (0, σ2)].

The treatment effect was assessed with regression analysis, using PROC GENMOD to evaluate linear, quadratic, and cubic effects, and PROC NLMIXED for the linear plateau (LP) model. The aim was to identify the optimal level of cookie residue inclusion in the broiler diet. The models were compared using the Akaike information criterion (AIC), considering the log of maximum likelihood (−2 log L) and the number of explanatory variables in the model. A lower AIC value indicates a better fit to the data.

The linear model is defined by


$$\begin{array}{l} E\left(Y\right)=\alpha +\beta x \end{array}$$

and the LP broken line by


$$\;E(Y)={\{}_{\;\theta \;ifx\;>\;x_{0}}^{\;\alpha \;+\text{ β}x\;if\;x\;\leq \;x_{0}\;}or,\;E(Y)={\{}_{\;\theta \;ifx\;\leq \;x_{0}}^{\;\alpha \;+\text{ β}x\;if\;x\;>\;x_{0}\;}\;$$

where E(Y) is the expected value of the dependent variable, *x* is the level of cookie residue inclusion, θ is the value at the plateau, α is the intercept, β is the slope of the line, and *x*_0_ is the level of cookie residue inclusion in the diet at the inflection point of the line.

## 3 Results and discussions

### 3.1 Characterization of cookie residue

The DM; CP; gross energy; EE, CF, and MM contents; density; and MGD are described in Table 4.

**Table 4:** Characterization of cookie residue (CR) used in the metabolism and performance assays.

**Variable (%)**	**Cookie residue**				**Corn, grain 7.86**
	**CR**	**(4)**	**(9)**	**(10)**	**(4)**
Dry matter	91.70	92.50	91.92	94.06	88.90
Crude protein	8.34	8.69	3.10	7.96	7.86
Gross energy, Kcal kg^−1^	4,333	4,341	5,232	4,734	3,901
Ether extract	1.240	0.828	2.280	1.175	0.381
Crude fiber	1.65	1.70	–	–	1.73
Mineral matter	0.141	0.131	0.278	0.173	0.111
Density, g ml^−1^	0.5826	–	–	–	–
MGD, μm	542.37	–	–	–	–

Mean geometric diameter (MGD); cookie residue (CR).

Based on the values obtained in the present study and data from the literature, the nutritional value of cookie residue seems to vary depending on the process it is subjected to and the ingredients used in its manufacture. Indeed, there may be differences in the chemical composition of the residue for studies conducted in Brazil and abroad. For example, Fasolin et al. (9) and Gutkoski et al. (10) evaluated the nutritional composition of cookies made with banana flour and oat flakes and reported a CP content of 4.54% and 18.29%, respectively, and an EE content of 1.89% and 4.85%, respectively. The values obtained from the present study are higher than those reported by Rostagno et al. (4) for corn. Thus, the residue has great nutritional and energy potential when compared to corn.

The MGD for this study is slightly larger than that found by Nunes et al. (11), namely 538.34 μm. Considering the classification of foods based on MGD—thick (MGD ≥ 832.7 mm), medium (MGD of 375.3 to 832.7 mm), or thin (MGD <375.7 mm) cookie residue is classified as a medium food.

### 3.2 Metabolism trial

The AMCDM, AMCCP, AMCGE, AME, and AMEn for cookie residue were 67.05%, 65.03%, 82.08%, 3,959, and 3,480 Kcal kg^−1^, respectively. Although the AMEn is lower than that found in the Brazilian Tables, namely, 4,010 Kcal kg^−1^ (4), it is higher than the calculated value for corn (3,364 Kcal kg^−1^).

The calculated AME and AMEn are similar to those reported by Costa (5), namely 3,959 and 3,480 Kcal kg^−1^, respectively, for cookie residue in a metabolism experiment with 16-day-old broilers when replacing 30% of the reference feed with cookie residue. Nunes et al. (12) reported AME and AMEn values of 4,480 and 4,339 Kcal kg^−1^, respectively, for cookie residue for broilers. These figures are noteworthy when compared with the values attributed to corn. In a test conducted with free-range chickens, Santos et al. (13) reported results for AME of 3,817 ± 201.80 Kcal kg^−1^ for salty cassava starch cookie residue and 3,578 ± 43.09 Kcal kg^−1^ for salty + sweet cassava starch cookie residue. These values indicate an energetic contribution from cookie residue when incorporated into diets for birds.

These results confirm the high energy value of cookie residue, making it a viable alternative to traditional energy sources such as corn in broiler diets. The obtained AMEn value (3,480 Kcal kg^−1^) suggests that the ingredient can significantly contribute to the energy density of the diet, potentially allowing for partial replacement of conventional ingredients without compromising performance. Furthermore, the consistency of results across different studies underscores the reliability and nutritional stability of this ingredient, despite variations in processing or composition. Therefore, cookie residue can be considered a strategic ingredient, especially in regions where its availability and cost-effectiveness align with sustainable and efficient poultry production practices.

### 3.3 Performance

Table 5 presents the performance variables of broilers fed diets containing different levels of cookie residue.

**Table 5:** Average performance values of broilers fed diets containing increasing levels of cookie residue.

**Variable**	**Inclusion levels (%)**							
	**0**	**10**	**20**	**30**	**40**	***P-*** **value**	**CV%**	**Reg**
	**Pre-starter phase (1–7 days)**							
Weight	48.89 ± 0.14	48.84 ± 0.12	48.88 ± 0.13	48.86 ± 0.11	48.89 ± 0.12	0.998	0.62	ns
FI	131.8 ± 2.36	135.6 ± 2.73	130.3 ± 3.09	130.5 ± 1.14	131.9 ± 2.90	0.595	4.72	ns
BWG	109.8 ± 2.52	116.8 ± 1.95	114.1 ± 3.20	118.3 ± 1.43	115.0 ± 2.41	0.147	5.07	ns
FC	1.203 ± 0.024	1.163 ± 0.031	1.143 ± 0.012	1.104 ± 0.015	1.147 ± 0.010	0.030	4.25	Q^a^
Weight 7 days	158.6 ± 2.49	165.6 ± 1.94	163.0 ± 3.32	167.2 ± 1.45	163.9 ± 2.46	0.160	3.61	ns
	**Starter phase (8–21 days)**							
FI	933.2 ± 14.4	942.4 ± 9.02	950.6 ± 4.93	926.8 ± 6.88	943.9 ± 8.83	0.012	2.44	ns
BWG	718.0 ± 11.1	744.2 ± 8.61	753.6 ± 4.69	750.4 ± 3.49	755.9 ± 7.59	0.012	2.51	Q^a^
FC	1.300 ± 0.011	1.267 ± 0.015	1.262 ± 0.012	1.235 ± 0.013	1.249 ± 0.009	0.006	2.35	Q^a^
Weight 21 days	876.7 ± 13.3	909.8 ± 8.26	916.5 ± 5.91	917.6 ± 3.50	919.8 ± 7.50	0.423	2.25	Q^a^
	**Growth phase (22–35 days)**							
FI	1,947 ± 22	1,920 ± 24	1,956 ± 38	1,933 ± 26	1,888 ± 45	0.995	4.10	ns
BWG	1,176 ± 10	1,177 ± 24	1,176 ± 35	1,181 ± 17	1,166 ± 28	0.329	5.10	ns
FC	1.656 ± 0.010	1.633 ± 0.023	1.667 ± 0.027	1.637 ± 0.010	1.619 ± 0.007	0.714	2.56	ns
Weight 35 days	2,053 ± 22	2,087 ± 18	2,092 ± 34	2,099 ± 16	2,086 ± 29	0.615	2.89	ns
	**Finisher phase (36–42 days)**							
FI	1,311 ± 23	1,324 ± 50	1,274 ± 50	1,242 ± 18	1,282 ± 51	0.798	7.81	ns
BWG	703.4 ± 20.5	729.0 ± 50.3	682.4 ± 31.4	683.3 ± 14.4	679.3 ± 34.6	0.891	11.51	ns
FC	1.868 ± 0.039	1.842 ± 0.088	1.874 ± 0.053	1.821 ± 0.036	1.894 ± 0.039	0.930	7.19	ns
Weight 42 days	2,707 ± 33	2,767 ± 66	2,726 ± 54	2,733 ± 28	2,717 ± 59	0.657	4.49	ns
	**Accumulative period (1–21 days)**							
FI	1,065 ± 15	1,078 ± 10	1,081 ± 7	1,057 ± 6	1,076 ± 11	0.460	2.35	ns
BWG	827.8 ± 13.3	860.9 ± 8.2	867.6 ± 5.8	868.7 ± 3.6	870.9 ± 7.5	0.006	2.37	Q^a^
FC	1.287 ± 0.010	1.253 ± 0.014	1.246 ± 0.009	1.217 ± 0.011	1.235 ± 0.007	0.001	2.09	Q^a^
	**Accumulative period (1–35 days)**							
FI	3,012 ± 26	2,998 ± 30	3,037 ± 32	2,990 ± 29	2,964 ± 48	0.646	2.78	ns
BWG	2,004 ± 22	2,038 ± 18	2,043 ± 34	2,050 ± 16	2,037 ± 29	0.001	2.96	ns
FC	1.503 ± 0.006	1.471 ± 0.009	1.487 ± 0.014	1.459 ± 0.004	1.455 ± 0.005	0.715	1.39	L^b^
	**Accumulative period (1–42 days)**							
FI	4,323 ± 47	4,323 ± 70	4,311 ± 80	4,233 ± 34	4,246 ± 90	0.787	3.84	ns
BWG	2,707 ± 33	2,767 ± 66	2,726 ± 54	2,733 ± 28	2,717 ± 59	0.930	4.49	ns
FC	1.597 ± 0.009	1.564 ± 0.002	1.582 ± 0.010	1.549 ± 0.008	1.563 ± 0.009	0.103	1.98	ns

CV%, Coefficient of variation; ns, not significant; FC, feed conversion; FI, feed intake; BWG, bodyweight gain; Reg, regression. ^a^Quadratic effect. ^b^Linear effect.

The variables that showed significant differences were subjected to regression analysis; their equations are presented in Table 6.

**Table 6:** Regression equations of performance variables of broilers fed increasing levels of cookie residue.

**Variable**	**Regression**
FC in the pre-starter phase, g/kg	Y = 0.1048 × 2 −5.8948x + 1,207 (R^2^ = 0.8368)
BWG in the starter phase, g	Y = −0.0385 × 2 + 2.3588x + 720.32 (R^2^ = 0.9279)
FC in the starter phase, g/kg	Y = 0.0512 × 2 −3.3825x + 1,299.5 (R^2^ = 0.9195)
FW in the starter phase, g	Y = −0.0482 × 2 + 2.8682x + 879.61 (R^2^ = 0.9392
BWG accumulative 1 to 21 days, g	Y = −0.0483 × 2 + 2.8709x + 830.73 (R^2^ = 0.9391)
BWG accumulative 1 to 21 days, g	Y = −0.0483 × 2 + 2.8709x + 830.73 (R^2^ = 0.9391)
FC accumulative 1 to 35 days, g/kg	Y = −1.0994 + 1,497.1 (R^2^ = 0.7349)

FC, Feed conversion; BWG, bodyweight gain; FW, final weight; g, grams; Kg, kilogram.

In the pre-starter phase, only the feed-to-gain ratio was significantly different, showing a quadratic effect. According to the data, the feed-to-gain ratio in the pre-starter phase improved as the level of cookie residue inclusion in the experimental diet increased. Inclusion of 28.12% BR produced the best result for the feed-to-gain ratio. Diets with increasing levels of cookie residue inclusion were superior to the reference diet regarding feed conversion for the pre-starter stage.

It is noteworthy that the physical form and heat treatment to which the feed is subjected improve nutrient utilization, leading to better digestibility and absorption. This nutrient digestibility relies on the feed type, processing method, heating time and temperature, moisture, particle size, and inclusion level in the feed (14). Longo et al. (15) stated that the digestibility of carbohydrates such as starch can be observed shortly after hatching through the action of pancreatic amylase, which can be seen on day 14 of incubation but shows greater activity after day 4 of life. The absorption of yolk-sac-associated carbohydrate intake at this stage is essential for the bird to reach its growth potential (16). Freitas et al. (17) reported that starch gelatinization increases its digestibility, which occurs through exposure to amylose and amylopectin, whose granules are destroyed by heat. Starch gelatinization, followed by particle size and feed density, contributes to better feed conversion at this stage due to improved starch availability and enzymatic action on the substrate (food bolus) and, consequently, improved nutrient digestibility.

For the starter phase (days 8–21), there were significant differences for feed conversion, weight gain, and final weight at day 21 of life; each parameter showed a quadratic effect. The optimal level of cookie residue inclusion for feed-to-gain ratio, weight gain, and final weight at day 21 was 30.63%, 33.03%, and 29.75%, respectively.

For the cumulative period of days 1–21, there was a quadratic effect for feed-to-gain ratio and weight gain. There was a plateau at 31.74% and 29.72% cookie residue inclusion, respectively, for the feed-to-gain ratio and weight gain. After these levels, the response to the inclusion of cookie residue was stable but remained higher than what was observed for broilers fed the corn–soybean meal diet. These results corroborate those found by Sakomura (18). They evaluated the effect of different levels of AME in the diet and found an improvement in weight gain and feed-to-gain ratio of birds with an increase in dietary energy, associated with fat supplementation and an increase in caloric density. An additional caloric effect consists of increasing the availability of nutrients and enhancing fat metabolism, which improves energy efficiency by increasing the net energy of the feed.

For the cumulative period of days 1–35, the feed-to-gain ratio showed a linear decreasing effect: as cookie residue inclusion in the diet increased, the feed-to-gain ratio of the birds improved relative to the reference diet. The best responses for the variables occurred at 30%−40% cookie residue inclusion, with the feed-to-gain ratio being better than that of broilers fed corn-soybean meal.

The results are similar to the study by Pereira (19), who evaluated the partial replacement of corn with residue from the biscuit industry in the production of mallards and found an improved feed-to-gain ratio. They reported that the biscuit industry could replace up to 30% of corn in the feed, thus reducing production costs.

These positive results may also be due to the type of starch present in BR and/or gelatinization. Indeed, cookie production involves fermentation to form a sponge phase, followed by conditioning at high temperatures, which leads to structural modifications of the starch. Gelatinization is characterized by the rupture of hydrogen bonds that stabilize the internal crystalline structure of the granule when starch is subjected to heating and humidity; this structural modification increases the susceptibility to amylolytic degradation of the substrate (20, 21). Moreover, the rate and extent of starch digestion in the body can be influenced by several factors that increase the susceptibility of starch to amylase digestion in the gastrointestinal tract (22).

As the cookie residue inclusion in the diet increased, the fat content gradually rose due to the EE content. The EE content of cookie residue (12.40%) is much higher than what Rostagno et al. (4) reported for corn (3.81%). Better use of the fat in cookie residue may underlie its improved energy efficiency. Ribeiro et al. (23) stated that an increase in fats in rations presents benefits such as increased nutritional density, reduced speed of passage of the digestive bolus, greater aggregation of particles (with fats acting as binders), reduced powderiness, improved palatability, and optimized absorption of fat-soluble vitamins.

Table 7 presents the blood parameters of the 42-day-old birds. Based on regression analysis, the reference diet and the treatment diet with 100 g/kg cookie residue showed a linear plateau effect for cholesterol and creatinine, respectively. None of the other parameters, including glucose, triglycerides, and urea, showed significant differences between the treatments. Piotrowoska et al. (24) stated that the triglyceride level in broilers normally decreases from the 2^nd^ week of life due to growth and mobilization for the formation of adipose tissue. The glucose levels in the present study are below the range considered normal for healthy birds (200–500 mg/dL) (25, 26). Café et al. (27) reported a blood glucose level of 309.17 mg/dL; they attributed the increase in glucose to stress during blood sampling.

**Table 7:** Blood parameters in 42-day-old broilers fed increasing levels of cookie residue.

**Variable**	**Levels (%)**							
	**0**	**10**	**20**	**30**	**40**	**CV%**	***P-*** **Value**	**MD**
TC mg/dL^−1^	102.6 ± 6.5	119.8 ± 10.1	138.3 ± 3.1^*^	132.1 ± 8.5^*^	132.1 ± 7.5^*^	14.69	0.019	LP
CR mg/dL^−1^	0.237 ± 0.006	0.270 ± 0.009^*^	0.264 ± 0.004^*^	0.275 ± 0.099^*^	0.268 ± 0.016^*^	6.29	0.034	LP
GL mg/dL^−1^	171.7 ± 14.3	181.2 ± 12.7	167.9 ± 7.7	172.3 ± 10.9	171.8 ± 9.5	15.94	0.939	ns
UR mmol/L^−1^	5.16 ± 0.47	5.72 ± 0.47	5.62 ± 0.29	6.16 ± 0.40	5.43 ± 0.39	18.3	0.893	ns
TR mg/dL^−1^	112.7 ± 11.7	109.5 ± 9.8	101.6 ± 4.1	114.9 ± 5.7	107.1 ± 7.2	17.78	0.521	ns

CV%, Coefficient of variation; MD, model; LP, linear plateau; ns, not significant; TC, total cholesterol; CR, creatinine; GL, glucose; UR, urea; TR, triglyceride. ^*^Significant difference (*p* < 0.05) in the level of inclusion compared with the reference diet.

Cholesterol and creatinine showed significant differences; therefore, they were subjected to regression analysis (Table 8).

**Table 8:** Probability levels were observed in the regression analysis for the effects of cookie residue in the diet on cholesterol and creatinine levels in 42-day-old broilers (non-fasting, *n* = 14 per treatment).

**Probability level**	**Cholesterol**	**Creatinine**	**Glucose**	**Urea**	**Triglyceride**
Treatment	0.0186	0.0341	0.9391	0.5214	0.8025
Linear	0.0053	0.0141	0.8208	0.4581	0.8215
Quadratic	0.0502	0.0310	0.9448	0.2163	0.6953
Cubic	0.8203	0.9310	0.6143	0.6402	0.5269
Regression deviation	0.3712	0.3211	0.5044	0.3517	0.3261
R2 quadratic	0.939	0.959	-	-	-
R2 linear plateau	0.972	0.987	-	-	-

The linear plateau effect offers better adjustment according to the comparative analysis of models using the Akaike information criterion.

The linear plateau effect for cholesterol is defined by adjusting the equation:


$$\;E(Y)={\{}_{\;134.38\;mg/dL,\;if\;x\;>\;18.48\;\%}^{\;102.63\;+\;1.717x\;,\;if\;x\;\leq \;18.48\;\%\;}\;$$

with an inflection point at the cookie residue inclusion level of 18.48%, reaching an estimated plateau value of 134.38 mg/dL. Cookie residue inclusion increased the cholesterol levels, indicating that the birds absorbed fats from the offered diets. Almeida et al. (28) observed a decrease in cholesterol levels in 21-day-old broilers, which resulted from the high energy demand associated with greater body development in birds at this age. Of note, serum cholesterol concentration can be influenced by age, the type of diet, and reproductive activity (29, 30). Although there were variations for the experimental diets relative to the reference diet, according to Schmidt et al. (25), plasma cholesterol concentrations for most birds range from 100 to 250 mg/dL, highlighting that the levels found in the present research are within normal limits.

The linear plateau effect for creatinine is defined by adjusting the equation:


$$\;E(Y)={\{}_{\;0.2693\;mg/dL,\;if\;x\;>\;20.39\;\%}^{\;0.2368\;+\;0.0016x,\;if\;x\;\leq \;20.39\;\%\;}$$

with an inflection point at the cookie residue inclusion level of 20.39%, reaching an estimated plateau value of 0.2693 mg/dL.

The creatinine levels found in the present study ranged from 0.2367 to 0.2745 mg/dL, corroborating the data reported by the Merck Manual (31) (0.16-0.41 mg/dL in chicken serum) and by Batina et al. (32) (0.48 mg/dL). The value is much lower than that reported by Barbosa et al. (33) (1.2–2.2 mg/dL in laying hens) and by Café et al. (27) (1.41 mg/dL when evaluating chickens raised in a thermoneutral environment). According to Kaneko et al. (34), the normal reference range is 0.1–0.4 mg/dL. Wyss and Kaddurah-Auok (35) emphasized that the blood creatinine level is directly proportional to muscle mass, age, and physical activity. Moreover, similar to other chemical components of the blood, it is influenced by the diet to which the animal is subjected. According to Barbosa et al. (33), creatinine has little diagnostic value for laying hens because creatine is excreted by the kidneys before being converted to creatinine. As a result, it has a low concentration in the serum.

Szabó et al. (36) found higher creatinine levels than those in the present study when evaluating the development of growing turkeys at day 3 of life (12.0 mg/dL) and at the end of the fattening period (10.6 mg/dL). Piotrowoska et al. (24) found a creatinine level of 5.02 mg/dL in Ross lineage chickens from the 6^th^ week of life onwards, which is the period of greatest activity in bird muscle growth. The literature reports that around 1.7% of creatine in the body is irreversibly converted to creatinine and excreted. Creatine is a central constituent in the energy metabolism of birds. Plant-based diets restrict the dietary intake of creatine and condition metabolism to act in favor of greater economy in the excretion of creatinine, which is the only way that broilers have to excrete creatine-related metabolites. Nunes et al. (37) evaluated more than 1,000 blood samples from 42-day-old broilers and established reference values for blood metabolites under different sample collection and handling conditions. The reference range for creatinine is close to that cited by Kaneko et al. (34), confirming that values up to 0.40 mg/dL are considered normal. Any comment on the creatinine level must be based on compliance with standard operating procedures for analysis, which presuppose reading values in the linearity range.

The carcass characteristics variables are presented in Table 9. The variables that showed a significant difference were subjected to regression analysis (Table 10).

**Table 9:** Carcass characteristics of 42-day-old broilers fed diets with increasing levels of cookie residue.

**Variable**	**Levels (%)**							
	**0**	**10**	**20**	**30**	**40**	***P-*** **Value**	**CV %**	**R**
**Carcass weight (g)**								
SW	2,661 ± 54	2,730 ± 73	2,755 ± 57	2,784 ± 80	2,673 ± 86	0.0002	6.39	ns
HC	2,047 ± 45	2,102 ± 54	2,118 ± 41	2,135 ± 55	2,045 ± 62	0.0005	6.07	ns
CC	2,035 ± 44	2,090 ± 53	2,109 ± 40	2,124 ± 55	2,034 ± 63	0.0004	6.08	ns
**Carcass yield (%)**								
HC	76.93 ± 0.55	76.98 ± 0.43	76.88 ± 0.21	76.73 ± 0.30	76.52 ± 0.37	0.1834	1.24	ns
CC	76.47 ± 0.56	76.55 ± 0.36	76.57 ± 0.27	76.31 ± 0.27	76.10 ± 0.33	0.3783	1.20	ns
**Weight of cuts (g)**								
BRT	76.93± 0.55	76.98± 0.43	76.88± 0.21	76.73± 0.30	76.52± 0.37	0.0049	6.35	ns
DR	272.17 ± 9.00	278.58 ± 9.46	280.50 ± 5.98	282.51 ± 7.65	271.30 ± 7.66	0.0001	7.11	ns
TH	585.33 ± 14.0	601.33 ± 20.4	606.25 ± 7.87	611.06 ± 14.9	587.73 ± 17.7	0.0001	7.46	ns
WI	190.75 ± 4.44	195.33 ± 3.69	197.33 ± 4.03	198.94 ± 5.39	189.86 ± 6.23	0.0005	6.10	ns
BA	496.25 ± 13.2	510.33 ± 16.0	515.25 ± 15.0	516.83 ± 16.7	495.30 ± 15.4	0.0004	7.40	ns
**Cutting yield (%)**								
BRT	37.47 ± 0.37	37.48 ± 0.34	37.45 ± 0.42	37.50 ± 0.27	37.41 ± 0.04	0.6991	2.07	ns
DR	13.36 ± 0.20	13.34 ± 0.24	13.31 ± 0.18	13.33 ± 0.32	13.35 ± 0.08	0.6760	4.01	ns
TH	28.76 ± 0.27	28.76 ± 0.33	28.78 ± 0.40	28.79 ± 0.23	28.90 ± 0.08	0.1286	4.57	ns
WI	9.377 ± 0.1228	9.360 ± 0.1013	9.354 ± 0.1203	9.375 ± 0.0722	9.333 ± 0.0369	0.1207	2.52	ns
BA	24.39 ± 0.42	24.40 ± 0.20	24.41 ± 0.49	24.33 ± 0.38	24.35 ± 0.04	0.1486	3.49	ns
**Viscera weight (g)**								
HE	11.73 ± 0.62	11.21 ± 0.59	10.26 ± 0.56	10.16 ± 0.55	10.14 ± 0.56	0.0278	14.31	ns
LI	47.20 ± 2.05	51.05 ± 2.30	51.30 ± 1.03	54.01 ± 3.85	52.88 ± 4.30	0.0801	14.13	ns
FG	51.18 ± 2.10	44.52 ± 1.79^*^	46.00 ± 1.88	43.25 ± 1.26^*^	39.31 ± 1.53^*^	0.0001	9.47	L
EG	38.64 ± 1.42	35.02 ± 1.55	34.87 ± 1.41	34.04 ± 1.41	31.25 ± 1.05^*^	0.0001	9.71	L
SLI	95.94 ± 3.12	94.00 ± 4.31	90.09 ± 1.84	92.81 ± 4.18	90.61 ± 3.63	0.0441	9.33	ns
AF	40.91 ± 4.08	46.59 ± 4.14	55.08 ± 3.67	60.16 ± 3.42	56.50 ± 4.54^*^	0.2563	18.84	LP
**Viscera yield (%)**								
HE	0.45 ± 0.01	0.44 ± 0.01	0.42 ± 0.01	0.44 ± 0.01	0.47 ± 0.02	0.4555	9.01	ns
LI	40.91 ± 4.08	46.59 ± 4.14	55.08 ± 3.67	60.16 ± 3.42	56.50 ± 4.54	0.0943	11.12	ns
FG	1.922 ± 0.071	1.641 ± 0.081^*^	1.672 ± 0.063	1.568 ± 0.068^*^	1.481 ± 0.079^*^	0.0092	10.75	LP
EG	1.452 ± 0.0488	1.284 ± 0.040^*^	1.265 ± 0.035^*^	1.232 ± 0.062^*^	1.172 ± 0.035^*^	0.0012	8.67	LP
SLI	3.611 ± 0.1347	3.442 ± 0.0890	3.273 ± 0.0456	3.331 ± 0.0952	3.388 ± 0.0729	0.3066	6.62	ns
AF	1.541 ± 0.162	1.706 ± 0.156	2.014 ± 0.156	2.164 ± 0.123^*^	2.117 ± 0.152^*^	0.7207	19.27	LP

CV%, Coefficient of variation; LP, linear plateau; ns, not significant; R, regression; SW, slaughter weight; HC, hot carcass; CC, cold carcass; BRT, breast; DR, drumstick; TH, thigh; WI, wing; BA, back; HE, heart; LI, liver; FG, full gizzard; EG, empty gizzard; SLI, small and large intestine; AF, abdominal fat. ^*^Differ from the control by the Dunnett test (*p* < 0.05).

**Table 10:** Regression equations for carcass variables of 42-day-old broilers fed increasing levels of cookie residue.

**Variables**	**Status**	**Equation**	**R^2^**	**Level**
Abdominal fat, g	Linear plateau^a^	y = 0.7083 x0 +40 if x0 ≤ 25.25 y = 57.88 if x0 > 25.25	0.9678	25.25
Empty gizzard, g	Linear^b^	y = −0.1575x + 37.914	0.8877	-
Abdominal fat, %	Linear plateau^c^	y = −0.02365 x0 + 1.5173 if x0 ≤ 26.35 y = 2.1962 if x0 > 26.35	0.9844	26.35
Empty gizzard, %	Linear plateau^d^	y = −0.00935 x0 + 1.4273 if x0 ≤ 24.09 y = 1.2021 if x0 > 24.09	0.8746	24.09

^a^*p* = 0.0025; ^b^*p* = 0.0008; ^c^*p* = 0.0349; ^d^*p* = 0.0196.

There was a linear decreasing effect for absolute weight (g/bird) in the empty gizzard. As cookie residue inclusion increased, the weight of the gizzard decreased compared with broilers fed the reference diet. The absolute weight of abdominal fat showed an outline effect, with a plateau at 25.25% cookie residue inclusion providing an increase in the amount of fat deposited. After this level, fat deposition stabilized, but even the highest level of cookie residue inclusion produced a higher absolute weight of abdominal fat compared with the reference diet.

The empty gizzard yield showed a broken line effect, with a plateau at 24.09% cookie residue inclusion. This is linked to the higher density associated with the lower MGD of the feed due to less work carried out by the gizzard. When birds are fed denser feed containing smaller particles, the gizzard reduces its activity, showing little motility and directly affecting the development of the organ. According to Amerah et al. (38), the development of the gastrointestinal tract in broilers, especially the gizzard, is influenced by the size of food particles because this organ plays an important role in digestion, acting as a crusher/mixer. Indeed, this muscular organ can exert a pressure that exceeds 585 kg/cm^2^ (21). Therefore, when birds receive feed with smaller particles, there can be a reduction in gizzard weight and enlargement of the proventriculus (39). The gizzard will act as a transit organ rather than a grinding organ (40).

The relative weight of abdominal fat showed a broken line effect with a plateau at 26.35% cookie residue inclusion. There was maximum fat deposition at this level, followed by stabilization. This finding agrees with the higher density and EE content of the experimental diets compared with the reference diet. Consistently, Barbosa et al. (41) evaluated the effect of energy on performance, carcass yield, and abdominal fat, and reported that the increase in energy in the feed is directly linked to the increase in the deposition of abdominal fat in birds.

Although this increase in abdominal fat may be interpreted as a physiological response to the higher energy intake, the values observed remain within the expected range for broiler chickens. Furthermore, this metabolic adaptation did not compromise the productive performance of the birds, even at inclusion levels of up to 40% cookie residue. As discussed by Liu et al. (42), the accumulation of abdominal fat is a common feature in broilers, primarily associated with the birds' genetic profile and their high feed conversion efficiency. The author also emphasizes that, although undesirable from a commercial standpoint, this fat deposition does not necessarily indicate a pathological condition but rather reflects an adaptation to a positive energy balance.

Therefore, the use of cookie residue as an alternative feed ingredient appears to be feasible and safe up to the 40% inclusion level, showing promising results in terms of performance. Nevertheless, the observed changes in parameters such as cholesterol and abdominal fat deposition highlight the importance of considering not only productive aspects but also metabolic responses, especially in formulations with higher energy density. Future studies may explore complementary nutritional strategies, such as the inclusion of additives that modulate lipid metabolism, aiming to optimize the energy utilization of the ingredient and minimize potential side effects.

## 4 Conclusion

The inclusion of up to 40% cookie residue in broiler diets proved to be a viable nutritional strategy, promoting improved feed conversion and greater efficiency compared to the standard corn and soybean meal-based diet. Notably, carcass yield was maintained, and serum biochemical parameters such as cholesterol and creatinine remained within the physiological ranges expected for broiler chickens. Although an increase in abdominal fat deposition was observed, this effect appears to reflect a physiological adaptation to the higher energy density of the diets rather than a sign of metabolic impairment. These findings support the use of cookie residue as a sustainable, safe, and effective alternative ingredient in broiler feed formulations.

## Data Availability

The original contributions presented in the study are included in the article/supplementary material, further inquiries can be directed to the corresponding author.
